# Understanding Consumer Acceptance of Genetically Modified Foods: A Theory of Planned Behavior–Brand Equity–Sociodemographic Interactions

**DOI:** 10.3390/foods15162918

**Published:** 2026-08-20

**Authors:** Latifa Attieh, Jorge De Andrés Sánchez, María Puelles Gallo, Mario Arias-Oliva

**Affiliations:** 1 Antonine School of Business (ASB), Antonine University, Baabda P.O. Box 40016, Lebanon; 2 Social and Business Research Laboratory, Department of Business Administration, Universitat Rovira i Virgili, 43204 Reus, Spain; jorge.deandres@urv.cat; 3 Department of Marketing, Universidad Complutense de Madrid, 28224 Pozuelo de Alarcón, Spain; mpuelles@ucm.es (M.P.G.); mario.arias@ucm.es (M.A.-O.)

**Keywords:** genetically modified foods, theory of planned behavior, brand equity, configurational analysis, fuzzy set qualitative comparative analysis

## Abstract

This study examines consumer acceptance of genetically modified foods (GMFs) by integrating the Theory of Planned Behavior (TPB), brand equity dimensions, and sociodemographic factors within a configurational framework. Drawing on a sample of 405 Spanish consumers, the research combines correlational analysis with fuzzy-set qualitative comparative analysis (fsQCA) to identify the causal paths leading to both purchase intention and purchase non-intention. The findings show that the traditional TPB constructs and brand equity dimensions are positively associated with purchase intention, whereas belonging to older generations is negatively associated with it. However, the configurational analysis reveals a more nuanced picture. Purchase intention is explained by four configurations, grouped into two main profiles: one based on favorable subjective norms among younger consumers, and another centered on the joint presence of brand trust and brand familiarity. In contrast, purchase non-intention is explained by seven configurations, mainly characterized by the absence of favorable TPB conditions and weak brand equity, especially lack of trust and familiarity. The results confirm the causal asymmetry between acceptance and rejection of GMFs, as the configurations leading to purchase intention are not the mirror image of those leading to purchase non-intention. These findings challenge the assumption that GMF acceptance and rejection are symmetrical phenomena, and demonstrate that brand trust and familiarity complement attitudinal factors and constitute relevant conditions in several configurations associated with purchase intention. From a managerial and policy perspective, the findings suggest that promoting GMFs’ acceptance requires not only improving attitudes and perceived social approval, but also fostering trustworthy and familiar brand environments that may contribute to reducing uncertainty, alongside credible scientific information and effective institutional oversight.

## 1. Introduction

There is growing interest in advances in biotechnology, particularly within the agri-food industry. At the same time, these advances have increased consumer concerns, including issues related to the safety, ethics, and environmental impacts of genetically modified organisms (GMOs) in food [1]. Moreover, consumers’ understanding of the technology behind genetically modified foods (GMFs) is characterized by a certain degree of ambiguity, due to factors such as neophobia [2], as well as fear and lack of knowledge [3,4]. This context makes the continued analysis of this phenomenon essential for marketing professionals, governments, policymakers, and firms seeking to commercialize this type of food. Undoubtedly, one of the most widely used models for analyzing consumer decision-making is the Theory of Planned Behavior (TPB) [5], which has been extensively applied across numerous domains, including food-related decisions and the acceptance of agricultural technologies [6,7], as well as in the specific case of GMF acceptance [8]. Substantial cross-national heterogeneity characterizes how consumers perceive and evaluate GMFs [9,10]. They are influenced by sociodemographic characteristics such as age, gender, and educational level [11,12], as well as by geographical and cultural factors [9,13,14,15]. Individual beliefs, perceived risks and benefits, and knowledge about genetic modification also shape consumer responses [16,17]. Perceived health risks [18] and environmental concerns [19] should also not be overlooked.

GMFs may reach consumers through supply chains comprising agricultural producers, processors, manufacturers, distributors, retailers, regulatory bodies, and final consumers. The length and complexity of food supply chains may vary according to the type of product and the number of stakeholders involved. Some products follow relatively direct production and distribution routes, whereas others involve additional stages of processing, certification, distribution, and retailing, which may increase information asymmetry and make credence attributes more difficult for consumers to assess [20]. These differences may affect transparency, traceability, and consumers’ ability to identify the origin and characteristics of GM ingredients. In this context, brands may operate as informational cues that reduce uncertainty and influence perceptions of familiarity and quality, particularly when consumers have limited knowledge of the technology or product [1,3,4,17]. These perceptions may also differ across sociodemographic groups, since age and gender have been associated with differences in attitudes, risk perceptions, and openness toward GMFs [8,11,12]. Thus, to complement the explanatory perspectives provided by the variables mentioned above, researchers have identified brand equity-related factors as additional sources of credibility and stability [20]. Indeed, branding can alleviate consumer skepticism toward GMOs [21]. Branding elements such as brand image and brand trust have the potential to provide value and positively influence consumer attitudes toward GMFs [22]. In [23] it is noted that, although an increase in food prices may negatively affect consumer utility, the presence of a brand may positively influence purchase behavior.

These considerations motivate the present study, which analyses GMF purchase intention (PI) through an integrative approach combining TPB and brand equity elements. Accordingly, the outcome examined is consumers’ stated behavioral intention to purchase rather than their actual purchasing behavior. This approach represents a first contribution, as it has been only partially explored in the literature. Wu et al. [24] proposed an integrated framework combining customer-based brand equity and TPB, suggesting that brand awareness and perceived quality positively influence brand trust, which in turn affects consumer purchase intentions. Rabbanee et al. [25] explored the factors influencing repurchase intentions and behavioral loyalty toward GMFs using the TPB model, highlighting the lack of a comprehensive understanding of how demographic variables, particularly gender and age, influence attitudes and repurchase intentions. The present study extends this literature by jointly considering distinct brand equity dimensions (brand trust, brand familiarity, brand image, and perceived quality) together with TPB and sociodemographic conditions. A second novelty of this study is that the integration of TPB with branding elements is carried out from a configurational perspective grounded in complexity theory [26]. According to this perspective, in economic and business phenomena, the paths leading to an outcome—which in this case is PI—are not unique, but emerge from different forms of interaction among antecedent variables [26]. Likewise, in this type of phenomenon, the causes leading to the presence and absence of the outcome are not usually symmetrical [27]. This approach also makes it possible to determine whether the different brand equity dimensions play distinct or interchangeable roles across alternative consumer configurations.

**Figure 1:**
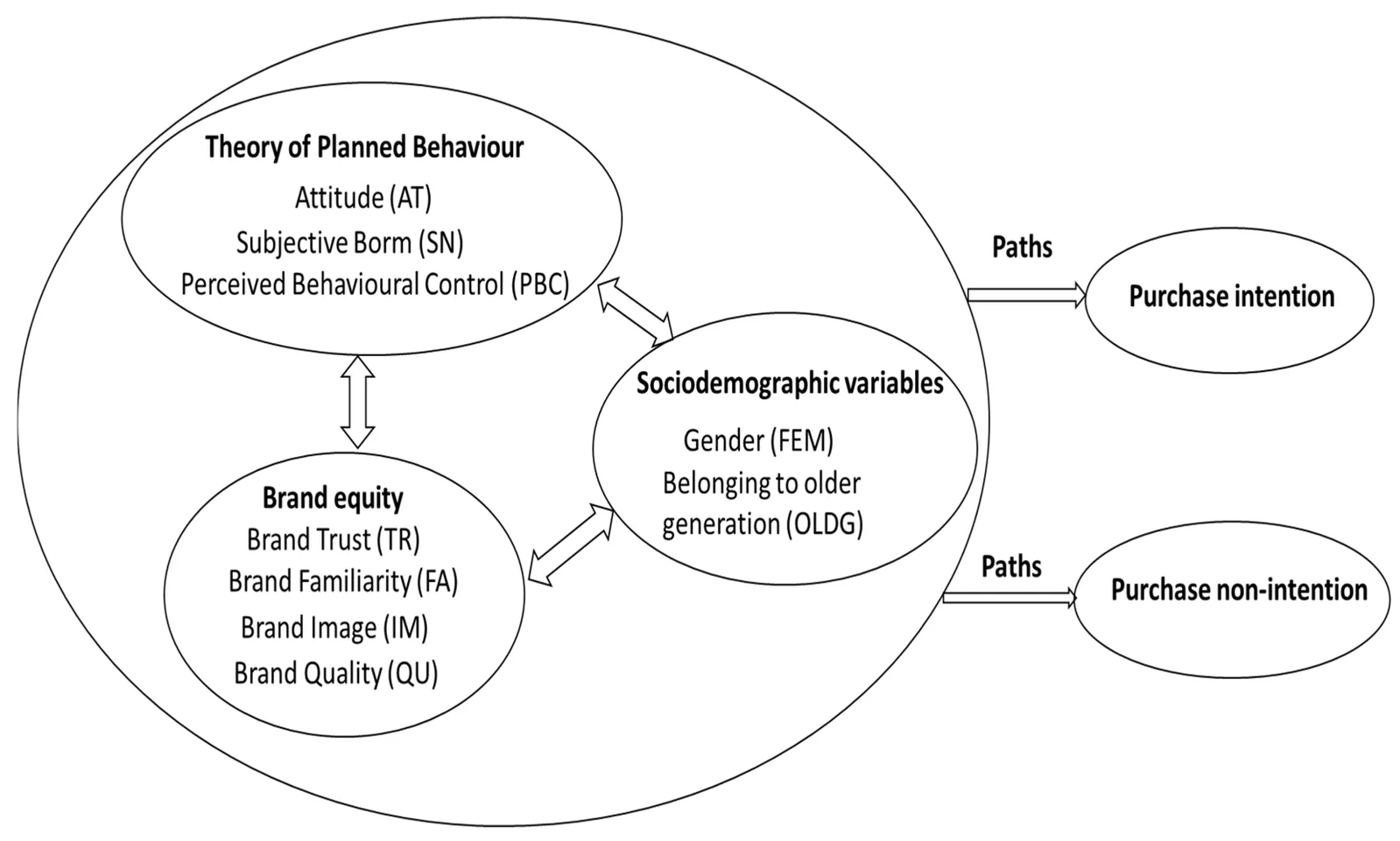
Conceptual Model.

The assumptions of complexity theory have repeatedly been shown to be particularly applicable to consumption-related phenomena [27,28]. To the best of our knowledge, the combination of a conceptual framework based on TPB and brand equity with an approach grounded in complexity theory has not yet been explored in the analysis of consumer behavior toward novel foods in general, nor, by extension, toward GMFs. Implement complexity theory, fuzzy-set qualitative comparative analysis (fsQCA) is a particularly suitable analytical tool, as it allows for the examination of multiple causality and asymmetry in the relationships among variables. Its suitability is also reflected in the growing use of this method in marketing and consumer behavior studies since the mid-2010s [29]. Thus, based on the conceptual framework shown in Figure 1 and on the considerations outlined above, this study seeks to answer the following research question:

RQ: How do the factors shown in Figure 1, which combine TPB elements, brand equity dimensions, and sociodemographic factors, interact to produce the conditions contained in the paths leading to GMF purchase intention and purchase non-intention?

## 2. Literature Review and Hypotheses Development

### 2.1. Integrating the Theory of Planned Behavior and Brand Equity to Explain GMF Purchase Intention

This study develops a configurational model that integrates elements of the TPB [5], brand equity constructs [30], and two sociodemographic variables commonly considered in studies on food acceptance: gender and generational membership [8]. Although TPB has proven effective in predicting behavior across various fields, some researchers suggest that including additional constructs could enhance the model’s predictive power [19,31,32]. In this study, as shown in Figure 1, we complement the TPB approach with brand equity. Brand equity facilitates consumers’ interpretation and processing of product information, increases confidence in purchase decisions, and contributes to their overall evaluation of the consumption experience [30]. Brand equity is generally understood as the additional value that consumers attribute to a brand when they are familiar with it and perceive it favorably [33,34]. Although several brand equity models exist, those proposed by [33,35] are among the most widely used in the literature [34,36,37]. Specifically, this study adopts four relevant dimensions of brand equity—brand trust, brand familiarity, brand image, and perceived quality—which are commonly regarded as essential for building strong consumer–brand relationships.

In this study, the term brand does not necessarily refer to a brand that consumers already associate specifically with GMFs. Rather, it denotes the corporate, manufacturer, or retail brand under which a genetically modified food product would be marketed. According to the customer-based brand equity perspective, consumers develop knowledge structures and associations that extend beyond the product itself and influence the evaluation of new offerings introduced under the same brand [33]. Consumers may therefore transfer pre-existing perceptions of the brand, such as trust, familiarity, image, and perceived quality, to a newly introduced GMF, even when they have limited awareness of brands currently offering such products. By integrating brand equity with TPB, marketing specialists can obtain a more nuanced understanding of the factors that drive consumers’ intentions and behaviors toward brands, thereby enabling the design of more targeted and effective marketing strategies [38]. It has been shown that factors associated with brand equity, such as trust and marketing communications, may help mitigate some negative perceptions and contribute to more favorable attitudes toward GMFs [39]. Branding elements such as associations, loyalty, and perceived quality may contribute to brand equity, which can in turn be linked to the components of TPB to better understand consumer behavior [40].

In the following subsections, we first develop correlational hypotheses on how TPB constructs, brand equity, and generational membership influence purchase intention. This correlational perspective will subsequently allow us, in light of the interrelationships among the different constructs, to formulate testable propositions from a configurational perspective that considers not only the sign of the relationships between variables, but also the asymmetry between the factors that may foster purchase intention and those that may inhibit it.

### 2.2. Correlational Hypotheses on Genetically Modified Food Purchase Intention from the Theory of Planned Behavior Perspective

The TPB, as adapted to consumer behavior, posits that purchase intention is driven by attitudes (AT), subjective norms (SN), and perceived behavioral control (PBC) [5]. This is a common approach for investigating consumer behavior toward GMFs [41,42,43]. Among the TPB constructs considered in the aforementioned studies, AT emerges as a particularly influential element in predicting behavior toward GMFs across highly diverse cultural contexts, including countries in Europe, America, and Asia [41,42,43,44,45,46]. Similarly, SN has shown significant relevance, which may be explained by the limited public knowledge of GMFs [47]. In the absence of sufficient knowledge, consumers tend to give greater weight, in their decision-making process regarding GMFs, to the opinions of trusted individuals whom they perceive as more informed and who approve of this type of food [8,46].

To a more limited extent, the influence of perceived behavioral control on the intention to consume GMFs has also been demonstrated in the literature [44,48]. Accordingly, [49] showed that participants exposed to conditions of lower perceived control perceived greater risks associated with genetically modified foods, which in turn reduced their willingness to purchase them. Therefore, we propose the following hypotheses:

**H1.**  *A favorable attitude toward GMFs is positively associated with purchase intention.*

**H2.**  *A favorable subjective norm toward GMFs is positively associated with purchase intention.*

**H3.**  *Greater perceived behavioral control over GMFs is positively associated with purchase intention.*

### 2.3. Correlational Hypotheses on Genetically Modified Food Purchase Intention from the Perspective of Brand Trust

Brand trust (TR) is defined by Morgan and Hunt [50] as the perceived reliability and integrity of a product. Trust in the entities that produce and regulate GMFs is a critical determinant of consumer acceptance [51]. As a construct, brand trust has been shown to influence perceptions and acceptance of GMFs [51,52]. When consumers trust a brand, they are more likely to perceive its GMF products as safe and beneficial, leading to more favorable attitudes and a greater tendency to purchase them [53]. Conversely, lack of brand trust may contribute to skepticism and resistance to GMFs, even when the product presents favorable intrinsic qualities [44]. In [39] it is suggested that consumer attitudes toward GMFs may be influenced by perceptions of product quality and reliability, which are integral components of brand equity. PBC may positively influence both AT and PI and may strengthen the relationship between these variables [54]. This helps explain why brand trust may be a particularly influential variable in purchase intention in the context of fresh foods [55]. Accordingly, we propose the following hypothesis:

**H4.**  *Brand trust in a brand that commercializes GMFs is positively correlated with purchase intention.*

Brand familiarity (FA) can be defined as the knowledge consumers have of a brand, derived from direct or indirect experiences [35]. In general, consumers use their past experiences with familiar brands as cues to form favorable or unfavorable attitudes, predispositions, evaluations, and purchase decisions regarding those brands [56,57]. According to [58], brand familiarity has shown notable effects on consumer attitudes. This means that, when consumers have a high level of familiarity with brand names, they are more likely to develop positive associations and favorable attitudes toward those brands, and to show a greater predisposition to consume their products. This finding is consistent with the observation that, during the COVID-19 pandemic, shoppers were more inclined to purchase familiar brands across different categories, as these brands reduced uncertainty and risk [59]. Thus, we propose the following hypothesis:

**H5.**  *Brand familiarity with a brand that commercializes GMFs is positively correlated with purchase intention.*

Brand image (IM), as a component of brand equity, refers to the perception of a brand in consumers’ minds [33]. Parris and Guzmén [60] (p. 224) offer a more recent definition of brand image as “the network of positive and negative mental associations that stakeholders form about a brand.” Brand image can have an important impact on how consumers think, feel, and form opinions about a given product [61]. Diamantopoulos et al. [62] examine the positive effect of country-of-origin image, product category image, and brand image associations on purchase intentions. In addition, Ref. [24] noted that brand image, brand personality, and brand associations, together with SN, act as antecedents of attitudes toward the brand, thereby influencing consumer intentions. Therefore, we propose the following hypothesis:

**H6.**  *A better brand image of a brand that commercializes GMFs is positively correlated with purchase intention.*

The relationship between perceived brand quality (QU) and AT is significant, as perceived quality acts as a critical determinant of consumer trust and acceptance. Ref. [63] suggests that, when perceived quality is high, it reduces perceived risks and reinforces positive attitudes, ultimately increasing intentions to consume GMFs. This association between perceived quality and positive attitudes toward GMFs was confirmed by [45]. Favorable perceptions of brand quality may further contribute to consumer confidence in GMFs. Consumers are more likely to develop favorable attitudes when brands associated with GMFs consistently offer high-quality products, fostering trust and reducing uncertainty about the risks associated with genetic modification [34]. In this regard, perceived brand quality may complement the attitudinal component of TPB and contribute to explaining GMF purchase intention. Consequently, we propose the following hypothesis:

**H7.**  *Higher perceived brand quality in a brand that commercializes GMFs is positively correlated with purchase intention.*

Accordingly, these constructs are analyzed separately because they capture conceptually distinct consumer perceptions and need not develop in parallel [30,33]. For example, a brand may be highly familiar because of repeated exposure while still being perceived as unreliable, associated with an unfavorable image, or judged to offer low-quality products. Similarly, consumers may trust a brand to deliver consistently on its promises while perceiving its products as basic rather than high quality [64]. Brand familiarity therefore reflects accumulated knowledge and recognition; brand trust, confidence in the brand’s reliability and integrity; brand image, the positive and negative associations attached to the brand; and perceived quality, consumers’ judgments of its overall excellence or superiority [65]. Aggregating these dimensions into a single higher-order construct could therefore obscure their distinct relationships with purchase intention and their potentially different roles in the configurational analysis.

### 2.4. Correlational Hypotheses on the Association Between Sociodemographic Variables and Genetically Modified Food Purchase Intention

The literature has repeatedly shown that age, or generational membership, and gender are relevant factors in explaining consumers’ predisposition toward novel foods such as GMFs [8]. Younger generations, who tend to be more open to technological advances, may show more favorable attitudes toward GMFs, especially when these products are marketed by a well-known and trusted brand [66,67]. In contrast, older consumers may be more skeptical due to their preference for traditional food sources and possible concerns about the long-term health effects of GMFs [68]. Gender differences may also influence PI. Some studies suggest that women’s decisions are more strongly shaped by food safety concerns and, therefore, they may display more negative dispositions or higher risk perceptions toward GMFs [53]. Therefore, age and gender are included in this study as variables that may help explain GMF purchase intention. Accordingly, we propose the following hypotheses:

**H8.**  *Being female is negatively associated with GMF purchase intention.*

**H9.**  *Belonging to older generations, namely Generation X and baby boomers, is negatively associated with GMF purchase intention.*

### 2.5. Development of Configurational Propositions for GMF Purchase Intention

Purchase intention and purchase non-intention do not result from a single trajectory, but from a variety of paths that are not usually symmetrical. These trajectories can be captured through a configurational approach implemented with fsQCA [27,29]. This analytical perspective has also proven useful in the food domain; for example, in the analysis of online purchases of fruit products [69]. The literature has shown that consumers’ relationship with new-generation foods cannot be reduced to a simple dichotomy between acceptance and rejection; rather, both positions involve differentiated paths leading to them. Among those willing to consume such products, it is possible to distinguish food neophiles, who actively seek gastronomic novelty out of curiosity or as part of their identity [70], from value-driven enthusiasts, whose acceptance is driven by ethical concerns related to animal welfare or environmental sustainability [71]. These are joined by conditional acceptors, whose judgement depends on the specific benefit offered by each novelty [9]. In an intermediate position are curious but cautious consumers, who are willing to try these products provided that sufficient safety guarantees or broad social validation exist. Szakály et al. [72] identify four consumer groups, ranging from adventurous and open-minded individuals to more selective profiles. This classification suggests that openness toward novel foods is distributed along a continuum rather than forming a strict dichotomy. Of course, intermediate categories may exist between these profiles.

Consumers who are reluctant to purchase GMFs do not constitute a homogeneous group either. It can be distinguished between rejection based on perceived health risk, which may potentially be modified through information; sensory-based rejection, which may be reduced through exposure and experience; and moral or identity-based rejection, linked to notions of naturalness and food authenticity, which is virtually unmoved by rational arguments [73]. These profiles are complemented by individuals reluctant due to institutional distrust, for whom the negative attitude does not stem from the food itself, but from a lack of trust in the scientific, regulatory, or industrial bodies that support it [74]. This plurality of profiles has direct implications for food communication and marketing, because each consumer group may require a different strategy. In this line, the present study seeks to identify profiles of individuals willing to purchase GMFs, as well as profiles of individuals inclined toward rejection, using the integration of TPB and brand equity as its conceptual basis and adopting an empirical perspective. Since our analysis is configurational, it is important to stress that TPB variables, as shown by its derived models, are not isolated dimensions but interact with one another. For example, it has been widely demonstrated that subjective norm and ease of use—the latter linked to perceived behavioral control—positively influence perceived usefulness [75].

Similarly, brand equity elements are interrelated variables [34]. The different dimensions of brand equity act jointly to shape consumers’ evaluations of a brand and, consequently, affect their attitudes and purchase intentions. This interrelationship is reflected, for instance, in the relationship between several dimensions of brand equity and the formation of brand trust. Ref. [22] reveals that when consumers hold a favorable image of a brand, they develop greater trust in it. A positive perception of brand quality also leads to higher levels of trust [76]. Likewise, there is empirical evidence of the positive influence of FA on PI, since FA tends to increase TR, reduce perceived risk, and make the brand more attractive [38]. As noted in Section 2.1, there are also cross-interactions between TPB constructs and brand equity constructs. Attitudes toward a behavior, such as purchasing GMFs, are influenced by perceptions of QU, especially when these are linked to established and trusted brands. Studies on brand equity in the context of food consumption state that QU, together with variables such as IM and TR, may play a relevant role in shaping attitudes toward branded products [77,78,79]. Similarly, Ref. [80] used a combination of branding elements and TPB to explain the determinants of green brand equity, highlighting the role of brand image and consumer attitudes. According to [24], a positive brand image may shape subjective norms by affecting how consumers perceive others’ opinions about consuming GMFs associated with that brand. This relationship between subjective norms and brand image has also been addressed in the literature, although in a different context by [81].

The preceding paragraphs suggest that both purchase intention and purchase non-intention may follow multiple paths, corresponding to different typologies, resulting from combinations of the presence or absence of attributes linked to TPB and brand equity, which the literature recognizes as interrelated constructs. Configurational hypotheses are formulated flexibly through propositions or tenets, which establish in general terms how the paths leading to both the presence and the absence of the phenomenon analyzed should be understood [82]. This approach also reflects that the presence and absence of the outcome, PI, are not symmetrical. Accordingly, we establish the following three propositions:

**Proposition** **1.** *All TPB and brand equity constructs constitute conditions in at least one of the paths for GMF purchase intention, and this outcome requires their presence more frequently than their absence. Sociodemographic variables appear as conditions in at least one of the paths for this outcome. The condition of not being female appears more frequently than that of being female, while the condition of not belonging to older generations is present in more configurations than belonging to them.*

**Proposition** **2.** *All TPB and brand equity constructs constitute conditions in at least one of the paths for GMF purchase non-intention, and this outcome requires their absence more frequently than their presence. Sociodemographic variables also appear as conditions in at least one of the paths for this outcome. The condition of being female appears more frequently than that of not being female, while the condition of belonging to older generations is present in more configurations than not belonging to them.*

**Proposition** **3.** *The paths leading to purchase intention and purchase non-intention are not symmetrical.*

## 3. Materials and Methods

### 3.1. Schematic Overview of the Survey Program

**Figure 2:**
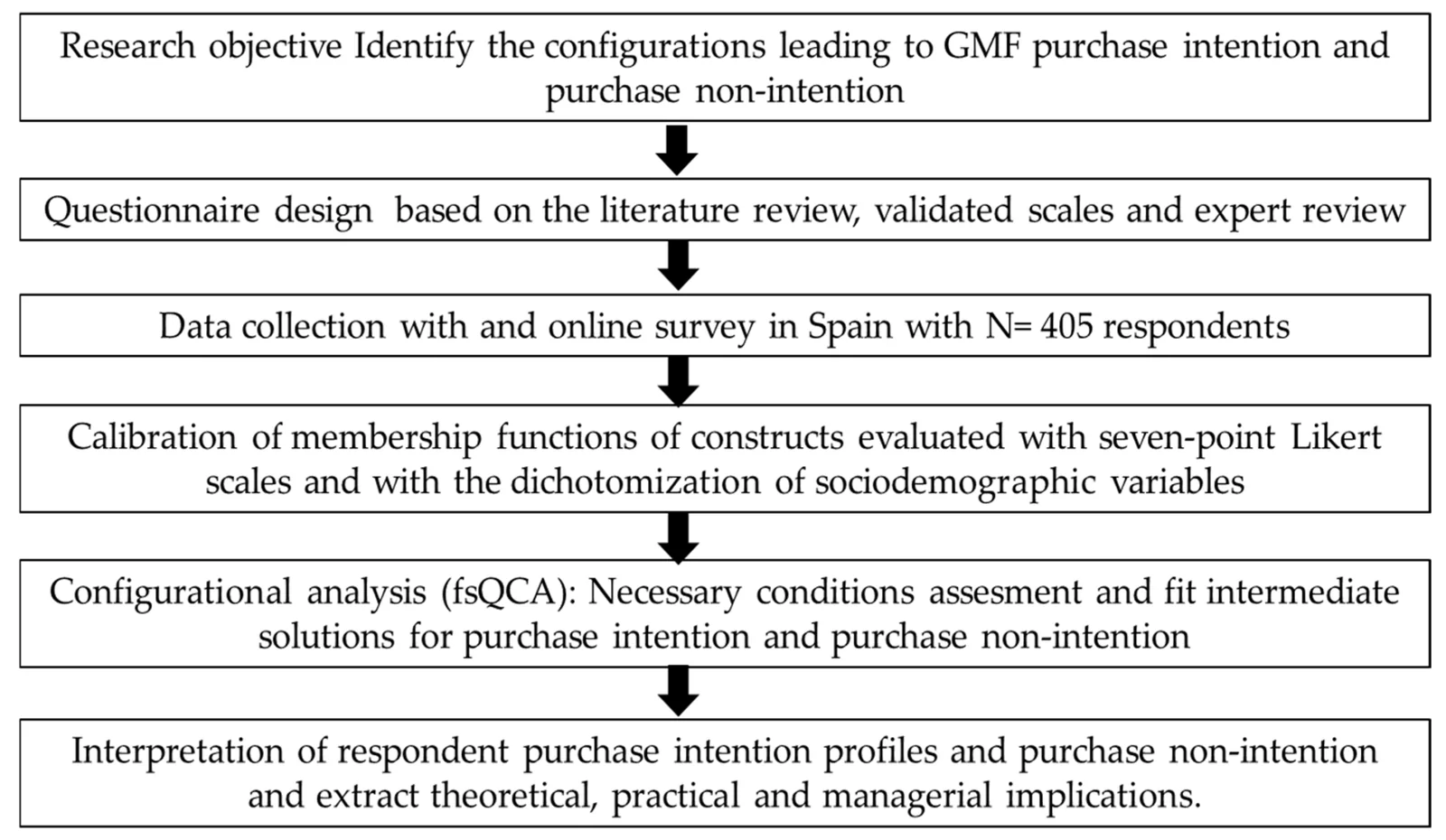
Schematic overview of the survey program and analytical procedure.

Figure 2 provides a schematic overview of the research design adopted in this study, from questionnaire development to the configurational interpretation of the results. The survey program was specifically designed to identify the combinations of behavioral, brand-related, and sociodemographic conditions associated with genetically modified food purchase intention and purchase non-intention. To ensure methodological rigor, the study employed previously validated measurement scales, expert review of the questionnaire, standardized data collection procedures, and complementary assessments of reliability, convergent validity, discriminant validity, and common method bias before conducting the configurational analysis. Finally, the configurational framework based on fsQCA was adopted because the objective of this study was not only to estimate the net effects of individual antecedents, but also to identify multiple, potentially asymmetric combinations of conditions leading to both purchase intention and purchase non-intention.

### 3.2. Sampling

The present study adopts a quantitative methodology to answer the research questions. In this regard, a structured, self-administered online survey, designed using Google Forms was distributed to a sample of 405 respondents aged 18 years and older residing in Spain. Data were collected between September 2024 and April 2025. Participants were recruited using non-probability convenience sampling. Recruitment focused on individuals with prior awareness of GMFs or an interest in food-related issues, as they were expected to understand the questionnaire and provide informed assessments. However, this procedure may introduce self-selection bias and overrepresent consumers who are more knowledgeable, involved, or willing to express opinions about GMFs, while underrepresenting less informed or less interested individuals. Consequently, although the responses may be more considered, the sample cannot be regarded as statistically representative of the general population, and the findings should therefore be generalized with caution [83].

The sample’s sociodemographic characteristics can be summarized as follows. Regarding gender, the sample consisted mainly of women, with 250 participants (61.73%), followed by 149 men (36.79%), while 6 respondents (1.48%) selected the “other” option or preferred not to answer. In terms of age, 118 respondents (29.14%) were under 25 years old, 91 (22.47%) were between 26 and 45 years old, 144 (35.56%) were between 46 and 60 years old, and 52 (12.84%) were aged 61 or older. Regarding employment status, 232 participants (57.28%) were employed, 22 (5.43%) were retired, 111 (27.41%) were students, and 40 (9.88%) reported other employment situations. Finally, in relation to monthly income, 41 participants (10.12%) reported earning less than €1000, 80 (19.75%) between €1001 and €2000, 75 (18.52%) between €2001 and €3000, and 103 (25.43%) €3001 or more, while 106 respondents (26.17%) preferred not to answer this question.

Women represented 61.7% of the sample, a distribution consistent with previous research showing that women are generally more likely to participate in surveys, including GMF-related studies [84]. Although this proportion differs from that of the general population, the imbalance is not substantial, and both gender categories are represented by a sufficient number of observations. Thus, the configurational results are unlikely to be an artefact of the underrepresentation of male respondents. Nevertheless, because convenience sampling was used, the prevalence and coverage of configurations involving gender should not be directly extrapolated to the Spanish population. A similar consideration applies to generational composition. Both younger and older participants are sufficiently represented in the sample, although the prevalence and coverage of configurations involving OLDG could differ in a population-based sample.

Employment status was not included as a condition in the configurational model because, unlike age or generational membership, it has not been consistently identified in the GMF acceptance literature as a particularly relevant determinant of consumer responses [4]. By contrast, generational differences have repeatedly been associated with openness toward food technologies and GMFs and were therefore incorporated into the conceptual model. Moreover, the two generational groups are almost evenly represented in the sample: 209 respondents belong to the younger group, comprising Generation Z and Millennials, whereas 196 belong to the older group, comprising Generation X and baby boomers. Consequently, the configurations involving OLDG are unlikely to result from a substantial numerical imbalance between the two generational categories.

### 3.3. Questionnaire and Measurement Model

At the beginning of the questionnaire, a brief and clear description of the study objectives was included, also explaining the voluntary nature of participation, the right to withdraw at any time, and the fact that all data would remain anonymous. The questionnaire was written in Spanish, as this was the language used in the context in which it was administered. It should be noted that an initial version of the questionnaire was reviewed by 11 university professors with diverse profiles related to food and GMFs, including professors of agri-food product marketing, nutrition specialists, and biotechnology experts. Their recommendations did not involve substantial changes to the questionnaire, but they did contribute to improving its readability.

All questionnaire items were derived from existing literature. The items were measured using a 7-point Likert scale, where 1 indicated “strongly disagree” and 7 indicated “strongly agree”. The wording of the items finally used in the analysis is presented in Table 1. Regarding the dependent variable, or outcome, namely purchase intention (PI), the items were adapted from [5]. For the items corresponding to the classic TPB variables, Ref. [85] was used as a reference for attitude (AT), [5] for subjective norms (SN) and for PBC. In turn, the items for the brand equity scales were adapted from [86,87,88].

### 3.4. Data Analysis

The RQ posed in the introduction was addressed using fsQCA, following the protocol proposed by [27]. Thus:

Step 1. The first step involved evaluating the measurement model, which was conducted as part of the correlational analysis. Pearson correlation analysis was used to assess whether the correlational hypotheses developed in the previous section could be supported with the available sample. This step was carried out using SmartPLS.

Step 2. The second step involved calibrating the membership functions for the latent and sociodemographic variables, generically denoted as mX for factor X.

Step 2.1. For the latent variables PI, AT, SN, PBC, TR, FA, IM, and QU, the neutral value of a scale ranging from 1 to 7 was set at 4. Accordingly, full non-membership (m_X_ = 0) was fixed at 2, the crossover point (m_X_ = 0.5) at 4, and full membership (m_X_ = 1) at 6. Values of m_X_ between 2 and 6 were linearly calibrated. This calibration procedure is commonly applied to seven-point scales, as shown in the methodological review by Pappas and Woodside [27].

Step 2.2. Generation and gender were modelled as dichotomous variables. Regarding generation, membership in older generations was captured through OLDG. Thus, m_OLDG_ = 1 was assigned to individuals older than 45 years, whereas m_OLDG_ = 0 was assigned to those younger than 45. At the time the survey was conducted, individuals aged 45 or older could be associated with Generation X or baby boomers, while younger respondents corresponded to millennials and Generation Z. The dichotomous operationalization preserves parsimony in the configurational analysis. This is relevant because the number of possible truth-table configurations increases rapidly as additional conditions are introduced in fsQCA [27]. Representing several age groups as separate sets would therefore increase the complexity of the truth table without necessarily providing a clearer generational distinction.

Regarding gender, the condition of being female was captured through FEM, so that m_FEM_ = 1 was assigned to women and m_FEM_ = 0 to all other cases.

The following steps were implemented using fsQCA 3.1 [89]:

Step 3. We examined whether the explanatory variables could act as necessary conditions for both PI and its absence. To do so, the presence of each variable X was represented through its membership score, m_X_, whereas its absence, expressed as ¬X, was calculated as m_¬X_ = 1 − m_X_. Following this logic, PI was represented by the membership function m_PI_, while purchase non-intention, expressed as ¬PI, was obtained as m_¬PI_ = 1 − m_PI_. Thus, purchase non-intention was not modeled as an independent psychological construct but simply as the fuzzy-set complement of purchase intention, following standard fsQCA set-theoretic logic.

Step 4. We then analyzed sufficient conditions. This analysis involved estimating both the intermediate and parsimonious solutions for PI and ¬PI. In fsQCA, these solutions are composed of different causal configurations, also referred to as recipes or prime implicants, which identify combinations of factors associated with the acceptance or rejection of GMFs. In other words, they represent the different paths leading to the outcome. The construction of the intermediate solution required establishing prior assumptions about the possible presence or absence of exogenous variables in PI and ¬PI, based on the hypotheses formulated in Section 2. The truth table was constructed using a frequency threshold of 1, a consistency threshold of 0.80, and a proportional reduction in inconsistency (PRI) threshold of 0.60.

Step 5. The results corresponding to the intermediate solution for PI and ¬PI were then presented and interpreted. Within these configurations, a distinction was made between core and peripheral conditions. Core conditions are those that appear simultaneously in both the intermediate and parsimonious solutions and are therefore considered to have greater causal relevance. Peripheral conditions, by contrast, appear only in the intermediate solution and are interpreted as elements with a secondary or complementary influence [90].

Step 6. The explanatory capacity of the intermediate solution and of each configuration obtained was assessed using consistency (cons) and coverage (cov) indicators. Consistency indicates the degree of robustness of each configuration, with values above 0.8 generally considered adequate, whereas coverage reflects its empirical relevance and may be interpreted as an approximation of effect size [26]. Consequently, the analysis of the different trajectories derived from the intermediate solution made it possible to test the propositions formulated in Section 2.

## 4. Results

### 4.1. Descriptive Overview of the Sample and Validation of the Measurement Model

In Table 1 can be checked that outcome (PI) and the constructs AT, TR, and FA have item means close to 4 or slightly above it, although some items fall below this value, without ever dropping below 3.5. In turn, the TPB construct SN and the brand equity constructs IM and QU show values below 4, but always above 3. The lowest-rated construct is PBC, whose mean does not reach 3. Overall, the item medians are generally around 4, except for PBC, where they range between 2 and 3. Although item normality is not a problem in fsQCA, it is worth noting that the Cramér–von Mises statistic indicates that the items do not follow a normal distribution.

Regarding scale validity and internal consistency, Table 2 shows that Cronbach’s alpha and the two composite reliability indicators suggest adequate internal consistency, as they present values above 0.7. Likewise, Table 1 shows that the factor loadings of the items on their respective constructs clearly exceed the threshold of 0.7. Common method bias was assessed using Harman’s single-factor test and a one-factor confirmatory factor analysis (CFA). In Harman’s test, the first principal component accounted for 46.7% of the total variance, remaining below the commonly used 50% threshold. For the CFA, all measurement items were specified to load onto a single latent factor. The model showed poor fit to the data (χ^2^(377) = 4512.58, *p* < 0.001; CFI = 0.650; TLI = 0.623; RMSEA = 0.165; SRMR = 0.089), indicating that a single common factor could not adequately account for the covariance among the measurement items. Therefore, common method bias is unlikely to represent a serious threat to the validity of the findings.

### 4.2. Correlation Matrix and Associated Measures Outcomes

Table 3 supports the discriminant validity of the scales. According to the Fornell–Larcker criterion, the square root of the AVE is higher in all cases than the absolute correlations between constructs [91], and none of these correlations exceeds the threshold of 0.85 [92]. This conclusion is further supported by the heterotrait–monotrait (HTMT) ratios, all of which remain below 0.85. The highest values are observed between brand familiarity and brand image (HTMT = 0.82), purchase intention and brand familiarity (HTMT = 0.81), and brand trust and brand familiarity (HTMT = 0.78). Moreover, the corresponding 95% bootstrap confidence intervals are [0.76, 0.87], [0.76, 0.86], and [0.72, 0.84], respectively, and none includes the value 1. These results confirm that the constructs are empirically distinguishable and support retaining them as separate dimensions rather than combining them into a higher-order construct [91].

The correlation analysis of the TPB constructs, brand equity dimensions, sociodemographic variables, and PI makes it possible to assess whether the average direction of the relationships proposed in Section 2 is supported. The results confirm that, in all cases, the direction of the relationships is consistent with the hypotheses. Regarding PI, the strongest positive association was observed for FA, with r = 0.77 (*p* < 0.01), followed by IM, with r = 0.63 (*p* < 0.01), and QU, with r = 0.61 (*p* < 0.01). AT, SN, and PBC each showed positive and statistically significant associations with PI, all with r = 0.56 (*p* < 0.01), whereas TR also exhibited a positive but weaker significant relationship (r = 0.24, *p* < 0.01). Regarding the sociodemographic variables, OLDG was negatively and significantly associated with PI (r = −0.28, *p* < 0.01), whereas the relationship with FEM was weak and not statistically significant (r = −0.08). Among the explanatory variables, the strongest positive associations were observed between FA and IM (r = 0.78, *p* < 0.01), FA and PBC (r = 0.75, *p* < 0.01), and IM and QU (r = 0.72, *p* < 0.01). These results indicate that, despite the existence of moderate-to-high correlations among some brand equity dimensions, the constructs capture related but distinct aspects of consumer perceptions, consistent with the discriminant validity assessment presented in Table 3.

### 4.3. Results of Fuzzy Set Qualitative Comparative Analysis

#### 4.3.1. Results About Necessary Condition Analysis

Table 4, which reports the necessity analysis, shows that, when considering a condition to be necessary if its consistency exceeds 0.90, no single condition can be regarded as necessary on its own. The condition that comes closest to this threshold for PI is the presence of FA, with a consistency of 0.85. Similarly, in explaining the absence of purchase intention, ¬PI, the conditions with the highest consistency levels are the absence of perceived behavioral control, ¬PBC, the absence of perceived image, ¬IM, and the absence of perceived quality, ¬QU. However, although all of them reach a consistency of 0.85, none reaches the 0.90 threshold. Although FA does not meet the conventional consistency threshold for necessity, its relatively high coverage of 0.82 indicates that the condition is empirically relevant and is not restricted to a small or highly specific subset of cases. Nevertheless, because its consistency remains below 0.90, FA should be interpreted as a prominent and broadly represented condition associated with purchase intention, rather than as a necessary or near-necessary condition. Likewise, for purchase non-intention, the relatively high consistency values of ¬PBC, ¬IM, and ¬QU are accompanied by moderate-to-high coverage values, suggesting that these conditions are empirically meaningful across a substantial proportion of the cases displaying the outcome. However, their failure to reach the conventional necessity threshold prevents interpreting them as necessary conditions.

In any case, Table 4 also shows that, for the variables positively correlated with PI, the consistency of their presence in explaining PI is higher than that of their absence. This pattern is observed for all TPB and brand equity variables. Regarding the sociodemographic variables, ¬OLDG—that is, belonging to younger generations—is more consistent as a condition for producing PI than OLDG, which is in line with expectations. By contrast, being female, FEM, presents greater consistency than not being female, ¬FEM, in producing PI, which may be interpreted as contradicting H8 and the sign observed in the correlation.

Symmetrically, in explaining ¬PI, the negation of the conditions associated with TPB and brand equity factors is more consistent than their presence. In the case of sociodemographic factors, belonging to older generations, namely Generation X and baby boomers, OLDG, is more consistent than not belonging to them, while being female, FEM, is also more consistent than not being female, ¬FEM.

#### 4.3.2. Results of Sufficient Condition Analysis

**Figure 3:**
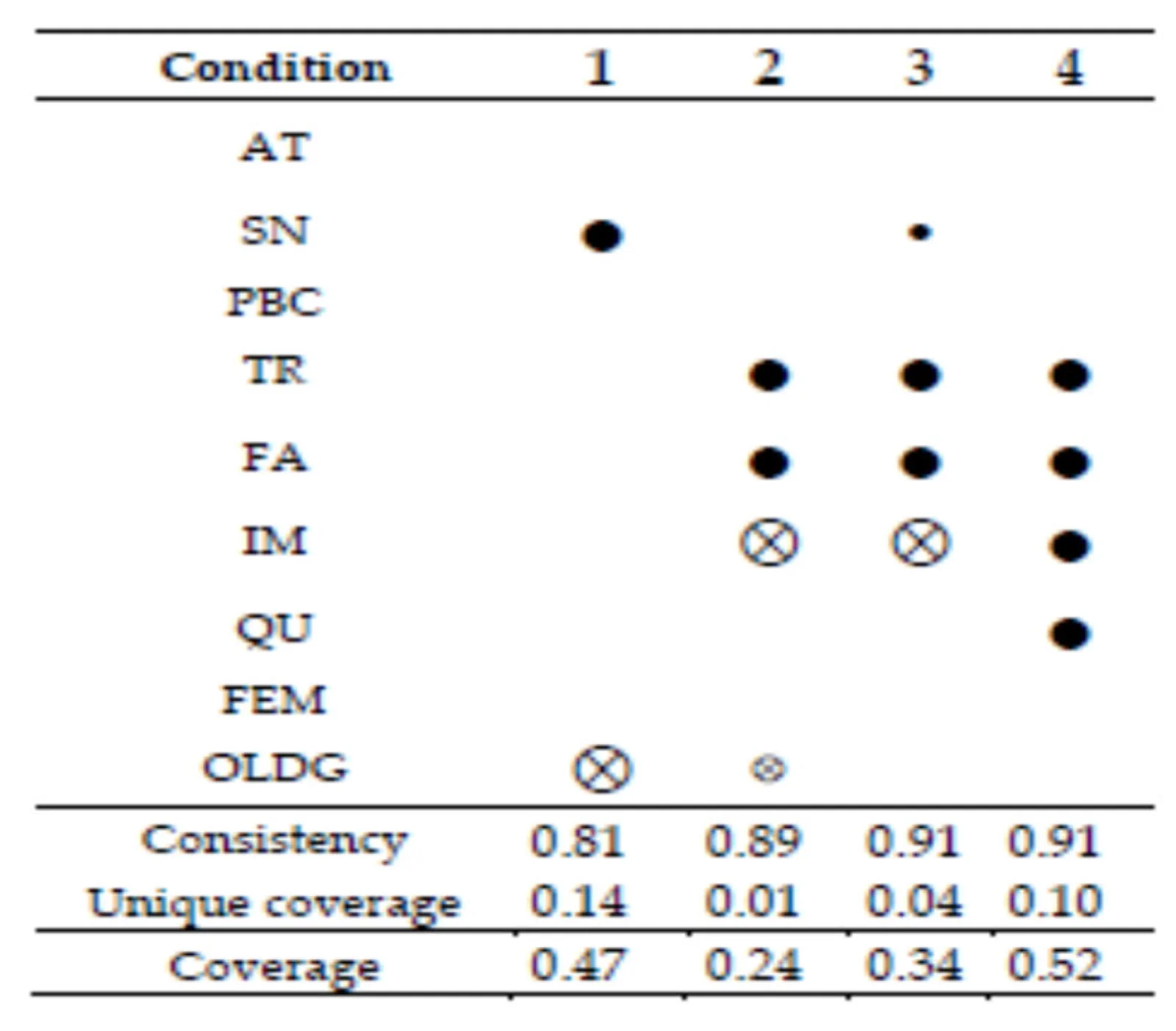
Paths of the intermediate solution of purchase intention GMF. Note: (a) • indicates the presence of a factor as a condition, ⊗ indicates the absence of a factor, and blank indicates no relevance in the prime implication. (b) Large circles represent core conditions and small circles represent peripheral conditions. (c) The coverage of the solution is 0.75, and the consistency is 0.82.

**Figure 4:**
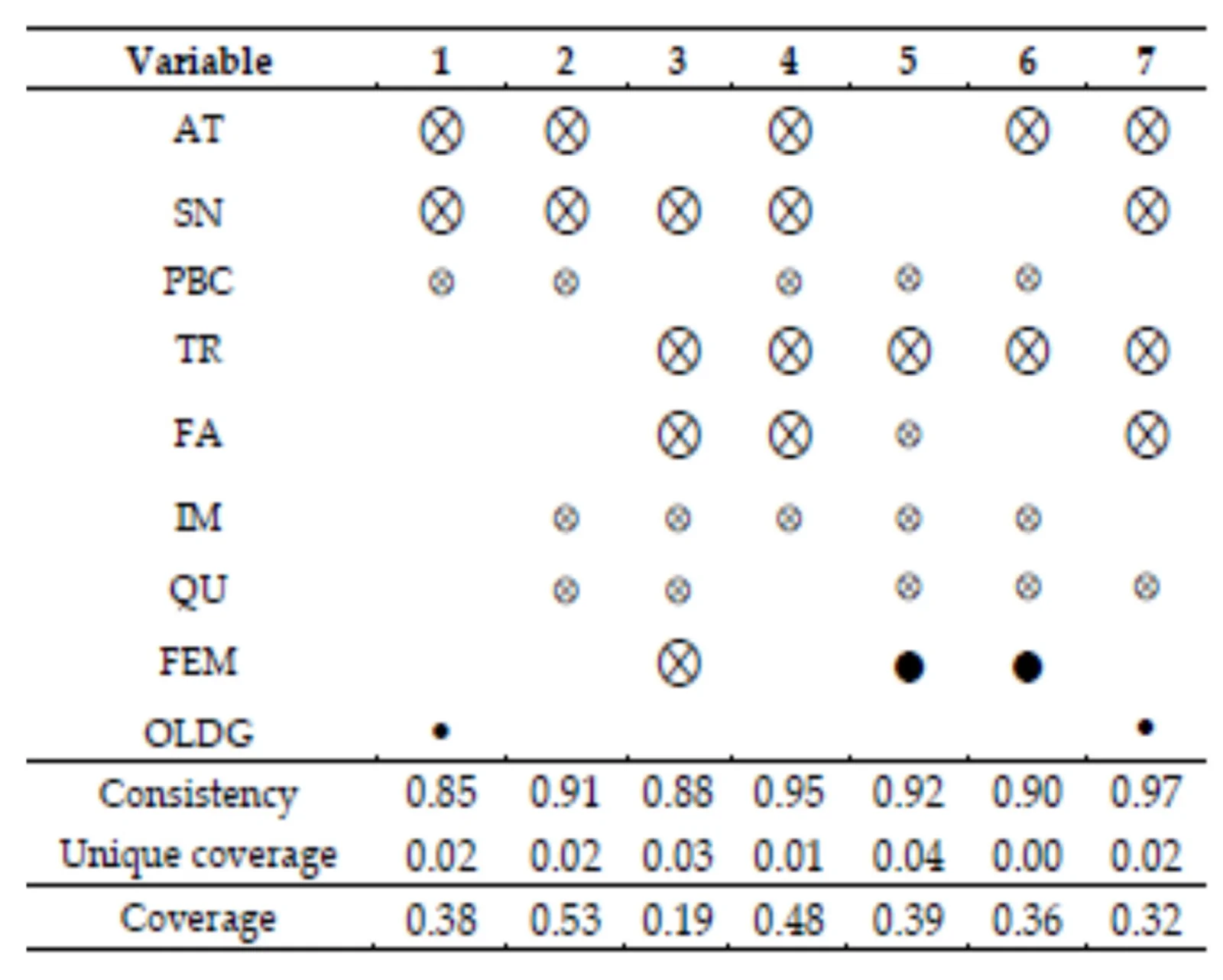
Intermediate solution for GMF purchase non-intention. Note: (a) • indicates the presence of a factor as a condition, ⊗ indicates the absence of a factor, and blank indicates no relevance in the prime implication. (b) Large circles represent core conditions and small circles represent peripheral conditions. (c) The coverage of the solution is 0.70, and the consistency is 0.84.

To facilitate interpretation, the individual configurational solutions in Figure 3 and Figure 4 were grouped into broader profiles according to their dominant common conditions. The grouping was performed only after obtaining the fsQCA intermediate solutions and was used exclusively to organize their substantive interpretation. Thus, this procedure does not modify the original fsQCA results but provides a more parsimonious synthesis of the configurations by highlighting the underlying patterns shared across several solutions, rather than discussing each path independently. Alternative strategies, such as reporting only configurations above a given coverage threshold, would have reduced the number of solutions at the cost of discarding relevant configurational information. Conversely, interpreting every individual path separately would have preserved the full diversity of the solutions but made it more difficult to identify the main behavioral profiles emerging from the analysis. For transparency, all original configurations are retained in the corresponding figures, while the proposed profiles serve only as an interpretative framework.

Figure 3 shows the configurations of the intermediate solution for PI, which presents a consistency of 0.82 and a notable coverage of 0.75. This solution includes four configurations, in which the acronym of factor X in uppercase indicates that it is a core condition, either X or ¬X depending on its presence or absence, whereas lowercase denotes a peripheral condition, that is, x or ¬x. Based on this, two groups of results can be distinguished.

(a) The first group identified consists of a path that includes only one TPB construct and one sociodemographic variable, SN•¬OLDG. In other words, PI would be explained by a positive evaluation of GMFs from the perspective of SN and would occur only among members of younger generations. In this configuration, brand equity variables would have no influence.

(b) The second group includes paths 2, 3, and 4, which can be algebraically expressed as TR•FA•(¬IM + sn•¬IM + IM•QU). They share the simultaneous presence of two brand equity constructs, trust and familiarity, as a common denominator. On this basis, three different configurations can be distinguished in which sociodemographic variables do not intervene and which essentially rely on core conditions associated with brand equity. It is noteworthy that the absence of perceived image, ¬IM, appears in two of them.

Regarding Proposition 1, it can be observed that only the statements referring to the presence of SN, TR, FA, and QU, as well as the absence of OLDG, are supported. On the one hand, although IM appears as a condition more often, it does so more frequently in its absence, in two configurations, than in its presence, in one configuration. On the other hand, AT, PBC, and gender do not appear as conditions in any of the configurations.

Figure 4 shows the intermediate solution for the absence of GMF purchase intention, ¬PI. The solution presents adequate consistency, 0.84, and an appreciable coverage of 0.70. It includes seven configurations, which can be grouped into three blocks.

(a) The first group includes paths 1, 2, and 4, whose algebraic expression can be written as ¬AT●¬SN●¬ pbc ●(oldg + ¬im●¬qu + ¬TR●¬FA●¬im). This group has a basis fundamentally associated with TPB, although PBC always appears as a peripheral condition. From this basis, the remaining conditions may be exclusively sociodemographic, as occurs with belonging to older generations, or may incorporate brand equity factors in their absence, although not necessarily as core conditions, as in the case of perceived image and perceived quality.

(b) The second group includes paths 3 and 7, which can be expressed as ¬SN●¬TR●¬FA●¬qu●(¬FEM + ¬AT●oldg). The basis of this group is given by the absence of subjective norm and by three brand equity-related conditions: the absence of perceived trust and familiarity as core conditions, together with the absence of perceived quality. On this basis, the configurations are completed either by the absence of being female, ¬FEM, or by the combination of absence of attitude and belonging to older generations.

(c) The third group includes paths 5 and 6, which can be written as ¬pbc●¬TR●¬im●¬qu●FEM●(¬fa + ¬AT). Although this basis includes constructs from the three types of factors analyzed, TPB, brand equity, and sociodemographic variables, the combination of absence of perceived trust and being female, FEM, stands out as core conditions. This group includes both those who additionally present the absence of familiarity with the brand that commercializes GMFs as a peripheral condition and those who show the absence of a favorable attitude toward these products.

We understand that the results fully support Proposition 2, since the TPB and brand equity variables appear in at least one configuration in their negated form, although PBC, IM, and QU always do so as peripheral conditions. Regarding sociodemographic variables, the results also show that OLDG participates as a condition in its presence, and that being female tends to appear as a condition in a greater number of configurations than its absence.

It is straightforward to verify that Proposition 3 is supported by the results: the paths leading to GMF purchase intention and those leading to its absence are not symmetrical, either in number, four versus seven, or in their internal configuration. For example, whereas neither AT nor PBC appears as a condition for PI, both are repeatedly present in ¬PI: AT as a core condition and PBC as a peripheral condition.

Moreover, whereas gender is not relevant for PI, in ¬PI being female participates in three configurations, once in its absence and twice in its presence. Likewise, although perceived image, IM, participates as a condition in three PI configurations, it does so with a contradictory sign—twice in its absence and once in its presence. By contrast, in ¬PI it appears in several configurations unequivocally in its absence, although always as a peripheral condition.

## 5. Discussion

### 5.1. Discussion of the Main Findings

This study seeks to identify the paths leading to both purchase intention and purchase non-intention, based on a model that combines the Theory of Planned Behavior (TPB) [5], a brand equity approach [35], and sociodemographic factors. The analysis is implemented using fsQCA, yielding solutions for both purchase intention and purchase non-intention with high levels of consistency and coverage. In line with [24,25], the results indicate that integrating TPB and brand equity constructs improves the explanatory capacity of TPB in the analysis of GMF acceptance. The measurement model demonstrates adequate internal consistency and convergent validity (refer to Table 1 and Table 2), while the discriminant validity assessment confirms that the constructs remain empirically distinguishable (refer to Table 3). Moreover, the correlation analysis reported in Table 3 supports all the hypotheses concerning purchase intention, except for the one referring to gender.

In Figure 3 and Figure 4, consumer profiles are identified for both purchase intention and purchase non-intention. Although this type of analysis has previously been applied in the literature to the study of acceptors [8,70,71] and rejectors [73,74], as well as by [72], this study adopts an empirical approach based on fsQCA rather than conventional cluster analysis, and with a different theoretical grounding. Figure 3 shows that two broad profiles of consumers with GMF purchase intention are identified. The first is characterized by the absence of brand equity involvement, with the presence of a positive subjective norm and belonging to younger generations being sufficient. The second profile is based on two core brand equity conditions—the presence of trust and familiarity—which combine with other core conditions from the brand equity approach. Regarding non-acceptors, three broad profiles are identified in Figure 4. The first is explained by the joint absence of TPB constructs, in which attitude and subjective norm act as core conditions. This basis is combined across different subprofiles with various sociodemographic and brand equity variables, which usually play the role of peripheral conditions. A second profile is characterized by the joint absence of brand trust and familiarity. This combination is accompanied either by not being female or by the joint absence of a favorable attitude and membership in an older generation. Finally, a third profile is based on the condition of being female combined with the absence of trust, which interacts either with the absence of familiarity or with the absence of a positive attitude.

The relatively limited role of PBC in Table 4 and Figure 3 and Figure 4 and it low evaluations in Table 1 deserve further consideration. Although PBC is a core component of the TPB, its influence might have been attenuated by the particular characteristics of the Spanish market. The PBC construct captured respondents’ perceived ability to make an informed decision about consuming GMFs and to identify these products in supermarkets. Although limited familiarity with GMFs could partly reduce perceived behavioral control, this explanation should be interpreted cautiously because the convenience sample included a relatively high proportion of individuals interested in health-related foods, who may be more familiar with food innovations than the general population. A more plausible explanation lies in the limited availability and visibility of GMFs intended for direct human consumption in Spain [8]. Consumers may therefore perceive that they have few opportunities to identify and purchase these products, regardless of their attitudes or intentions. Under these circumstances, perceived behavioral control may primarily reflect restricted market accessibility rather than consumers’ confidence in their own ability to consume GMFs. This suggests that avoiding such profiles requires not only reinforcing subjective norm, but also improving attitude and, to a lesser extent, perceived behavioral control.

Beyond the satisfactory discriminant validity indicated by the HTMT ratios in Table 3, the configurational results shown in Figure 3 and Figure 4 provide further support for treating TR, FA, IM, and QU as separate constructs rather than as a single higher-order factor. The acceptance paths do not incorporate these dimensions uniformly: TR and FA appear jointly in several configurations, whereas IM is present alongside them in some paths but absent in others, and QU appears only selectively. The rejection paths reinforce this interpretation, since IM and QU do not emerge as core conditions and TR and FA do not always operate jointly; some configurations include the joint absence of TR and FA, whereas others require only the absence of TR. These patterns show that the four dimensions may operate independently and even in opposite directions depending on the configurational context. Therefore, aggregating them into a single construct could obscure meaningful differences in their roles in PI and ¬PI.

### 5.2. Theoretical and Conceptual Implications

The combination of both TPB [5] and a brand equity groundwork [35] makes it possible to obtain robust solutions for explaining both purchase intention and non-intention GMFs. In this regard, the configurational analysis provides deeper insights that complement the correlational analysis. The latter shows that the direction of the relationships between TPB variables, brand equity dimensions, and purchase intention is, in general, as expected. It is also observed that generational membership is significant, whereas gender is not. Nevertheless, the configurational analysis allows for a more nuanced interpretation. Positive associations, such as those related to attitude, appear to be more relevant because their absence contributes to rejection. However, the presence of a favorable attitude does not necessarily generate purchase intention. This asymmetric finding implies that a favorable attitude may not be sufficient to generate an intention to purchase GMFs, but an unfavorable attitude can lead to purchase non-intention. This finding aligns with the principle of negativity bias, according to which negative information, experiences, and evaluations generally exert a stronger influence on judgement and behavior than equally intense positive ones [93]. Similarly, Ref. [94] differentiate between negative potency and negativity dominance, implying that negative stimuli are often more potent and influential than corresponding positive stimuli and may dominate an overall evaluation when positive and negative considerations are combined. Such a finding is particularly relevant in the context of GMFs, where consumers may rely more heavily on negative signals, including environmental consequences, unnaturalness, and safety and health concerns, than on positive outcomes when forming purchase intentions. In this regard, past literature on GMF acceptance has revealed that perceived risks, knowledge, trust, and perceived benefits jointly shape attitudes and purchase intentions, while limited trust and heightened risk perceptions may undermine consumers’ willingness to purchase GMFs [51,95]. Hence, it may not be sufficient to provide consumers with information on the benefits of GMFs, especially when negative evaluations are rooted in anxiety, moral concerns, naturalness beliefs, or distrust of institutions [96].

The reported asymmetry may also reflect a bias in consumers’ perceptions of GMF-related risks. When knowledge is limited or unbalanced, uncertain or unfamiliar hazards may be evaluated more severely than their expected benefits. Hwang and Nam [1] found that consumers who overestimated their knowledge of GMFs exhibited higher perceived risk, lower perceived benefit, and lower purchase intention; exposure to predominantly negative information also increased the likelihood of belonging to this group. Similarly, previous research indicates that consumers frequently perceive GMFs as involving greater risks than benefits, even when scientific assessments have not identified greater hazards than those associated with comparable conventional foods [8,47]. This gap between perceived and assessed risk may be reinforced by media narratives, unfamiliarity, institutional distrust, and labeling cues that consumers interpret as warnings. Recent evidence on gene-edited foods further shows that risk perceptions depend on the technology, product, information provided, and institutional context, with gene-edited products generally receiving more favorable evaluations than conventional GMFs [4]. Therefore, the greater configurational relevance of unfavorable attitudes and weak trust in explaining purchase non-intention may reflect not only negativity bias, but also a tendency to overweight uncertain or salient risks when evaluating GMFs.

Conceptually, these results reinforce the need to distinguish between the mechanisms generating acceptance and those producing rejection, rather than assuming that they are symmetrical opposites. They also illustrate the value of configurational approaches for identifying combinations of conditions that would remain hidden in models based exclusively on net effects. Similar to attitude, gender plays an asymmetric role in our study. Although gender does not appear as a relevant condition in the paths associated with acceptance, it does emerge in several profiles associated with purchase non-intention when combined with unfavorable behavioral and brand-related conditions. This result is partly supported by existing literature. For instance, Ref. [17] reported no significant gender differences in relation to attitudes toward GMFs. Similarly, Ref. [66] asserted that research investigating the role of gender in the acceptance of GMFs has yielded mixed results, with some studies reporting lower female acceptance while others have found higher female acceptance of GMFs. Thus, gender does not appear to explain acceptance, but it could play a conditional role in certain rejection trajectories. Thus, sociodemographic variables should be interpreted as contextual conditions whose relevance depends on their combination with behavioral and brand-related factors, rather than as direct determinants of GMF acceptance or rejection. More generally, the results support the integration of behavioral, brand-related, and sociodemographic conditions within the same configurational framework.

The findings also contribute to the conceptualization of brand equity in the context of controversial food technologies. Trust and familiarity do not operate merely as interchangeable manifestations of a global brand equity construct. Instead, they form part of specific configurations leading to acceptance, whereas their absence characterizes several paths associated with rejection. This supports treating brand familiarity, trust, image, and perceived quality as distinct conditions whose relevance depends on how they combine with TPB and sociodemographic variables. Likewise, TPB appears to make a limited contribution to explaining purchase intention, but a much more relevant contribution to explaining its absence. The main rejection profile is characterized by the joint absence of the three TPB constructs. This result suggests that TPB conditions may be particularly useful for understanding why consumers reject GMFs, whereas brand-related conditions play a more prominent role in several acceptance configurations. Consequently, integrating TPB and brand equity does not merely add predictors to the model; it reveals that the relative importance of each framework differs across acceptance and rejection pathways.

### 5.3. Managerial and Policy Implications

From a practical perspective, the configurational results suggest that interventions should be linked to the specific acceptance and rejection profiles identified, rather than applying a single strategy to all consumers. The findings therefore support differentiated managerial and policy actions.

Among younger generations, the brand seems to have limited relevance, whereas SN emerges as a key condition. In other words, when consumers see that important others, social groups, institutions, or trusted communities accept GMFs, they may be less dependent on familiar or trusted brands when forming purchase intentions. This finding aligns with the TPB, according to which subjective norm reflects the perceived social pressure to perform or avoid a behavior [5]. This can be particularly relevant in the context of GMFs, where consumers may have little experience or technical knowledge, and social approval can emerge as a useful cue [8]. Thus, for younger consumers, their acceptance of GMFs may increase when such foods are endorsed by people or institutions they find credible and trusted. This is especially relevant for younger consumers, whose behavior can be influenced by peer networks, digital communities, and social media environments in which attitudes and practices are rapidly shared and normalized [97,98].

In this case, SN can be strengthened through regulatory and informational public policies that increase the perceived social legitimacy of their consumption. These measures include clear and traceable labelling, institutional information campaigns based on scientific evidence, accessible dissemination of safety assessments and authorization procedures, as well as the involvement of health authorities, scientists, and consumer associations in public communication. Such actions may help consumers perceive that socially relevant actors consider GMF consumption to be acceptable and informed.

Communication through digital communities and partnerships with credible science communicators or influencers may also be particularly appropriate for this younger, subjective-norm-driven profile, provided that these actors are perceived as knowledgeable and independent rather than as purely commercial endorsers. For this profile, strengthening credible social endorsement may therefore be more effective than relying primarily on conventional brand advertising.

On the other hand, the second acceptance profile is structured around the combination of trust and familiarity. This suggests that, within the context examined, the commercialization of GMFs may benefit from the involvement of established food companies with high awareness and strong brand equity, compared with launches by unfamiliar firms or brands competing mainly on price. For this profile, firms could introduce GMFs through familiar product lines, maintain consistency between GMF-related claims and the brand’s established identity, and provide readily accessible information about ingredients, production methods, safety controls, and traceability. Independent certification, visible traceability systems, transparent labelling, and opportunities for consumers to become familiar with the product may further reduce uncertainty and reinforce trust. Nevertheless, brand credibility should be viewed as complementary to transparent scientific communication, regulatory safeguards, and institutional trust, rather than as a substitute for them [9,10]. This interpretation is reinforced by the identification of a clear low purchase-intention profile characterized precisely by the absence of trust and familiarity toward brands, or even by the sole absence of trust in profiles associated with potential female consumers. Trust is especially relevant, since its presence is included as a relevant condition in several configurations associated with purchase intention, whereas its absence constitutes a core condition in configurations of purchase non-intention. In this sense, consumers seem to transfer to brands the same requirements they project onto foods, namely safety, coherence, transparency, traceability, and honesty [99], even though these characteristics would, in principle, be attributable to the product rather than to the brand.

Firms should therefore avoid relying on broad reputational claims and instead provide verifiable evidence supporting these attributes. However, when the absence of trust forms part of a rejection configuration, brand-sponsored communication alone may be insufficient. In these cases, third-party certification, external audits, independent scientific validation, and communication involving regulatory authorities, health agencies, or consumer associations may be more credible than messages issued exclusively by the firm. This suggests that dealing with purchase non-intention profiles requires not only reinforcing SN, but also improving attitude and, to a lesser extent, PBC. These rejection profiles therefore call primarily for coordinated institutional and regulatory actions rather than for brand-building measures alone. Measures aimed at improving AT may be similar to those designed to strengthen subjective norm and may include providing more information, communicating evidence on the potential environmental benefits of GMFs, such as lower requirements for certain resources or greater resistance under extreme conditions, as well as disseminating their possible nutritional or taste improvements compared with conventional foods.

Such information should be balanced and should address consumers’ specific concerns regarding health, environmental consequences, naturalness, morality, and uncertainty rather than focusing exclusively on product benefits. Public authorities, scientists, and consumer organizations may be particularly important in these rejection profiles because they can provide information perceived as more independent than corporate communication. PBC may be strengthened through clear and easily understandable labelling, accessible information on how GMFs can be identified, transparent explanations of authorization and monitoring procedures, and greater visibility of the available product alternatives. These actions may improve consumers’ ability to recognize, evaluate, and choose GMFs rather than leaving them with the perception that they lack relevant information or control over the decision.

Overall, the results support a differentiated approach. Social endorsement and credible digital communication appear more suitable for the younger profile driven by subjective norm. Brand-based initiatives, product familiarity, traceability, and certification are more appropriate for the trust-and-familiarity acceptance profile. By contrast, institutional transparency, clear labelling, independent risk communication, and measures that improve consumers’ ability to identify and evaluate GMFs are especially relevant for the principal rejection profiles.

## 6. Conclusions

### 6.1. Main Takeaways

This study provides evidence that the acceptance of GMFs cannot be adequately explained through isolated net effects, but rather requires a configurational perspective that captures the combined influence of TPB, brand equity and sociodemographic factors. The results show that purchase intention and purchase non-intention are produced by different causal paths, confirming the relevance of causal asymmetry in consumer responses to GMFs. For purchase intention, two main profiles emerge. The first is mainly driven by a favorable subjective norm among younger consumers, suggesting that social approval and perceived legitimacy may be sufficient to foster acceptance in this group. The second is characterized by brand equity conditions, particularly the joint presence of brand trust and brand familiarity, which appear as central conditions in several acceptance paths. Thus, established and trusted brands may contribute to reducing uncertainty toward GMFs, particularly when their communication is supported by credible scientific information and effective regulatory oversight.

In contrast, purchase non-intention is explained by a broader and more complex set of configurations. These paths are mainly characterized by the absence of favorable TPB conditions and by weak brand equity, especially lack of trust and familiarity. Gender and generation are more relevant in rejection than in acceptance profiles. Overall, the findings indicate that fostering GMF acceptance requires not only improving attitudes and perceived social approval, but also building trustworthy, familiar and credible brand environments.

### 6.2. Limitations and Future Research

This study has several limitations that should be acknowledged and that open avenues for future research. First, the empirical analysis is based on a non-probabilistic convenience sample of Spanish consumers interested in food-related topics. Although this sample is appropriate for exploring reflective opinions on GMFs, the findings should not be generalized to the entire Spanish population or to other cultural contexts without caution. Future studies should replicate the analysis using probabilistic or quota-based samples and compare countries with different regulatory frameworks, cultural attitudes toward biotechnology, and levels of market exposure to GMFs. Such cross-cultural comparisons would also make it possible to assess whether the configurations identified in this study remain stable across different institutional, regulatory, and sociocultural environments. The assessment relies on stated purchase intention rather than actual purchase behavior. This is especially relevant in the case of GMFs, where consumers may express openness or rejection in survey contexts but behave differently in real market situations. Future research could combine survey data with experimental designs, choice experiments, or real purchase data to assess whether the identified configurations also predict behavior. Field experiments and discrete choice studies could also examine how price, labeling, product origin, and the communication of scientific information affect actual consumer decisions.

Future studies may also include additional antecedents, such as perceived risk, perceived naturalness, environmental concern, food technology neophobia, trust in public institutions, knowledge about genetic modification, or perceived benefits for health and sustainability. It would also be useful to distinguish between objective and subjective knowledge, as consumers’ actual understanding of GMFs may differ from how informed they believe themselves to be. Likewise, future configurational models could incorporate trust in science, ethical and moral concerns, food values, and confidence in regulatory and labeling systems. Moreover, operationalizing ¬PI as the fuzzy-set complement of PI does not distinguish between active rejection, indifference, and ambivalence. Respondents with intermediate membership scores may reflect uncertainty or mixed evaluations rather than a clear absence of purchase intention. Future research could therefore measure rejection, indifference, and ambivalence as distinct outcomes and examine whether they produce different configurational pathways.

This research adopts a cross-sectional design, which limits the ability to examine how consumer configurations evolve over time. Longitudinal studies could explore whether familiarity, trust, and subjective norms change as consumers receive more information, as GMFs become more visible in the market, or as public debate around biotechnology evolves. They could also determine whether the identified configurations remain stable throughout different stages of consumer learning, regulatory change, and market diffusion.

The calibration of the fuzzy sets was based on theoretically justified thresholds derived from the standard interpretation of a seven-point Likert scale, following the procedure recommended in [27]. Specifically, the selected anchors were used to define full non-membership, the crossover point, and full membership, after which intermediate values were converted into membership scores using a standard calibration function widely applied in the fsQCA literature. Nevertheless, calibration remains an open methodological issue. Future research could assess the robustness of the findings using alternative Likert-based anchor structures or other procedures for deriving membership functions, such as data-driven approaches based on principal component analysis [100]. Likewise, although fsQCA identifies necessary and sufficient set-theoretic relationships, it is descriptive rather than inferential and does not establish temporal causal direction [101]. Therefore, given the cross-sectional design, the configurations should be interpreted as cross-case regularities associated with PI and ¬PI, while reciprocal relationships between the outcome and some attitudinal or brand-related conditions cannot be excluded.

Future research should avoid treating GMFs as a homogeneous product category. Consumer responses may differ according to the type of food, the organism involved, the production technology used, and the specific benefit offered, such as improved nutrition, reduced pesticide use, greater environmental sustainability, longer shelf life, or lower prices. Comparisons between transgenic and gene-edited foods, as well as between plant- and animal-based applications, would provide a more nuanced understanding of the conditions underlying purchase and purchase non-intention.

## Figures and Tables

**Table 1:** Wording and descriptive statistics of items used in this paper.

Item	Mean	Median	SD	CvM	FL
Purchase intention (PI)					
PI1 = I would be willing to try a GMF if it were endorsed by a well-known brand.	3.96	4	1.93	1.08	0.97
PI2 = If a brand I trust launched GMFs, I would consider adding it to my shopping basket.	4.00	4	1.87	1.06	0.97
Attitude (AT)					
AT1 = I am interested in trying foods that include technological innovations.	3.81	4	2.00	1.17	0.93
AT2 = In general, I feel favorable toward GMFs.	4.00	4	2.02	1.33	0.93
Subjective norm (SN)					
SN1 = If some GMFs are approved by the government, I will have no doubts about consuming them.	3.99	4	1.94	1.43	0.82
SN2 = If people close to me started consuming GMFs, I would be more willing to try them.	3.74	4	1.94	1.22	0.93
Perceived behavioral control (PBC)					
PBC1 = I possess sufficient information to make a decision regarding GMF consumption.	2.99	3	1.81	2.35	0.94
PBC2 = If I wanted to, I could easily identify GMFs in supermarkets.	2.62	2	1.76	3.74	0.84
Brand trust (TR)					
TR1 = I trust that brands offering GMFs deliver on their promises.	4.01	4	1.82	1.08	0.79
TR2 = I am convinced that my preferred brands are truthful regarding their product information	3.96	4	1.66	1.18	0.9
TR3 = I trust the accuracy of the information that food brands provide about GMFs.	3.90	4	1.64	1.17	0.92
TR4 = I consider my preferred brands to be dependable and committed to safeguarding consumer welfare	3.94	4	1.59	1.31	0.92
TR5 = I am confident that my preferred brands adhere to applicable health and safety standards	4.51	5	1.68	1.36	0.85
TR6 = I believe my preferred brands operate with consumers’ best interests in mind and maintain transparency	3.80	4	1.68	1.16	0.87
Brand familiarity (FA)					
FA1 = I feel/would feel safe buying GMFs from brands I know.	4.14	4	1.83	1.05	0.92
FA2 = I expect the quality of GMFs is high in familiar brands.	4.23	4	1.82	1.15	0.93
FA3 = I trust that these brands will launch GMFs that are good.	4.29	4	1.78	1.05	0.92
FA4 = If these brands make a promise about a GMF, it is most likely true.	3.84	4	1.76	1.02	0.91
FA5 = Familiar brands make me feel confident when buying GMFs.	3.90	4	1.83	1.06	0.92
Brand Image (IM)					
IM1 = Brands that market GMFs seem trustworthy to me.	3.68	4	1.61	1.22	0.91
IM2 = I associate brands with GMFs with good-quality products.	3.53	4	1.68	1.16	0.92
IM3 = My opinion about brands offering GMFs is positive.	3.60	4	1.69	1.16	0.94
IM4 = I believe that brands offering GMFs are socially and environmentally responsible.	3.71	4	1.71	1.06	0.86
IM5 = I think that brands offering GMFs care about consumers’ health.	3.59	4	1.68	1.11	0.9
IM6 = Brands offering GMFs have a good reputation among consumers.	3.49	4	1.56	1.23	0.83
Brand quality (QU)					
QU1 = GMF is tastier than conventionally grown food.	3.41	4	1.55	1.70	0.77
QU2 = By eating GMF, I am eating healthier food.	3.29	3	1.54	1.60	0.94
QU3 = GMF is good for my family.	3.36	4	1.52	1.62	0.95
QU4 = GMF is produced in an environmentally friendly manner.	3.59	4	1.60	1.17	0.86

Notes: SD = Standard deviation. CvM denotes the Cramér–von Mises statistic and, in all items, its value leads to rejecting the normality of the distribution function. FL = factor loading.

**Table 2:** Measures of internal consistency and convergent reliability of scales.

Construct	C_α	ρ_a	ρ_c	AVE
PI	0.93	0.93	0.97	0.94
AT	0.85	0.85	0.93	0.87
SN	0.72	0.82	0.87	0.77
PBC	0.74	0.84	0.88	0.79
TR	0.94	0.95	0.95	0.77
FA	0.95	0.96	0.96	0.84
IM	0.95	0.96	0.96	0.80
QU	0.90	0.92	0.93	0.78

C_α = Cronbach’s alpha; ρ_a = Dijkstra–Henseler’s rho; ρ_c = composite reliability coefficient; AVE = average variance extracted.

**Table 3:** Correlation matrix, square root of AVEs and heterotrait-monotrait ratios.

	PI	AT	SN	PBC	TR	FA	IM	QU	FEM	OLDG
**PI**	0.97	0.64	0.67	0.28	0.58	0.81	0.67	0.67	NA	NA
**AT**	0.56 **	0.93	0.62	0.44	0.46	0.60	0.62	0.66	NA	NA
**SN**	0.56 **	0.50 **	0.88	0.27	0.47	0.61	0.57	0.53	NA	NA
**PBC**	0.56 **	0.42 **	0.41 **	0.88	0.26	0.31	0.38	0.35	NA	NA
**TR**	0.24 **	0.36 **	0.22 **	0.24 **	0.89	0.78	0.69	0.56	NA	NA
**FA**	0.77 **	0.54 **	0.53 **	0.75 **	0.27 **	0.92	0.82	0.70	NA	NA
**IM**	0.63 **	0.56 **	0.49 **	0.67 **	0.32 **	0.78 **	0.89	0.77	NA	NA
**QU**	0.61 **	0.57 **	0.45 **	0.53 **	0.28 **	0.65 **	0.72 **	0.88	NA	NA
**FEM**	−0.08	−0.10 *	−0.03	−0.02	−0.09	−0.02	−0.02	−0.05	1	NA
**OLDG**	−0.28 **	−0.40 **	−0.25 **	−0.09	−0.11 *	−0.23 **	−0.24 **	−0.26 **	0.06	1

Note: The main diagonal reports the square roots of the AVEs. The lower triangular section presents the Pearson correlations, where “**” denotes significance at the 1% level and “*” denotes significance at the 5% level. The upper triangular section reports the heterotrait–monotrait (HTMT) ratios.

**Table 4:** Necessary condition analysis.

Purchase Intention (PI)			Purchase Non-Intention (¬PI)		
**Condition**	**Consistency**	**Coverage**	**Condition**	**Consistency**	**C** **overage**
AT	0.74	0.76	AT	0.42	0.44
SN	0.74	0.77	SN	0.43	0.45
PBC	0.38	0.72	PBC	0.29	0.55
TR	0.78	0.76	TR	0.50	0.49
FA	0.85	0.82	FA	0.44	0.42
IM	0.70	0.83	IM	0.39	0.47
QU	0.64	0.84	QU	0.37	0.49
FEM	0.59	0.48	FEM	0.65	0.52
OLDG	0.39	0.40	OLDG	0.58	0.60
**Condition**	**Consistency**	**Coverage**	**Condition**	**Consistency**	**Coverage**
¬AT	0.45	0.44	¬AT	0.77	0.74
¬SN	0.46	0.45	¬SN	0.78	0.75
¬PBC	0.76	0.52	¬PBC	0.85	0.58
¬TR	0.48	0.49	¬TR	0.75	0.77
¬FA	0.40	0.42	¬FA	0.81	0.85
¬IM	0.55	0.48	¬IM	0.86	0.74
¬QU	0.61	0.49	¬QU	0.88	0.71
¬FEM	0.41	0.54	¬FEM	0.35	0.46
¬OLDG	0.61	0.59	¬OLDG	0.42	0.41

## Data Availability

The original contributions presented in the study are included in the article, further inquiries can be directed to the corresponding author.

## Abbreviations

The following abbreviations are used in this manuscript:

GMF	Genetically modified food
GMO	Genetically modified organism
TPB	Theory of Planned Behavior
TR	Brand Trust
QU	Perceived Quality
PI	Purchase Intention
AT	Attitudes
SN	Subjective Norms
PBC	Perceived Behavioral Control
FA	Brand Familiarity
IM	Brand Image
fsQCA	Fuzzy-set Qualitative Comparative Analysis
OLDG	Older Generation
FEM	Female
¬PI	Purchase nonintention
CvM	Cramér–von Mises statistic
AVE	Average Variance Extracted
